# MiR-449a regulates caprine endometrial stromal cell apoptosis and endometrial receptivity

**DOI:** 10.1038/s41598-017-12451-y

**Published:** 2017-09-25

**Authors:** Xiaopeng An, Xiaorui Liu, Lei Zhang, Junze Liu, Xinyan Zhao, Kaiwen Chen, Haidong Ma, Guang Li, Binyun Cao, Yuxuan Song

**Affiliations:** 1 College of Animal Science and Technology, Northwest A&F University, Yangling, Shaanxi 712100 P.R. China; 2 Northwest A&F University of Hospital, Northwest A&F University, Yangling, Shaanxi 712100 P.R. China

## Abstract

In this study, an RT-qPCR analysis showed that the expression levels of miR-449a in the pre-receptive endometrium were lower compared to the receptive endometrium, which is consistent with previous sequencing data (previous investigations). To detect the role of miR-449a in endometrial receptivity, we transfected caprine endometrial stromal cells (ESCs) cultured *in vitro* with miR-449a mimics. The results revealed that miR-449a decreased the mRNA and protein levels of LGR4 by targeting its 3′-untranslated region. The miR-449a mimics significantly reduced the G_1_ cell population from 52.56% (mimic NC) to 42.19% with a concordant increase in the G_2_ and S cell populations from 47.44% (mimic NC) to 57.81%, suggesting that miR-449a caused ESC cell cycle arrest. In addition, the number of apoptotic cells was significantly higher in ESCs transfected with miR-449a mimics (*P* < 0.05) than in ESCs transfected with mimic NC. *In vivo*, rich pinopodes were observed on the endometrial surface in the miR-449a agomir group compared with the miR-449a antagomir group. The results of hematoxylin-eosin staining showed that endometrial thickness was significantly increased in the miR-449a agomir group compared with the miR-449a antagomir group. These results suggest that miR-449a could enhance endometrial receptivity.

## Introduction

Implantation of the blastocysts into the maternal uterus is a crucial step in mammalian reproduction^1–3^, and successful implantation requires a receptive endometrium (RE)^4,5^, which cannot be separated from stromal cells^6,7^. Stromal cells represent a major cellular component of the endometrium that is subject to tight hormonal regulation; these cells express appreciable levels of estrogen and progesterone receptors and undergo different morphological changes upon hormonal stimulation^8^. Furthermore, stromal cells at the site of the implanting blastocyst undergo decidualization to provide nutrition to the developing embryo, protecting it from maternal immune responses upon invasion of the blastocyst into the endometrium^9^. However, embryo implantation in ruminants is non-invasive and does not involve stromal decidualization^10,11^. In addition, little information about stromal cells during embryo implantation in the goat is available.

MicroRNAs (miRNAs) are naturally occurring post-transcriptional regulatory molecules that potentially play crucial roles in endometrial receptivity^12^. Ponsuksili *et al*. (2014) identified significant miRNAs (miR-3902–3p, miR-1825, miR-H14–3p, miR-885–3p, miR-504–3p, and miR-186), miRNA-mRNA pairs, and functional networks that are associated with the state of pregnancy at Day 28 as a parameter of endometrial receptivity^13^. MiR-125b suppresses endometrial epithelial cell migration and invasion by down-regulating matrix metallopeptidase 26, which may restrain embryo attachment and subsequent invasion of the endometrium^14^. Our previous study indicated that miR-449a is the most differentially expressed miRNA, showing a 113.2-fold increase in the RE compared with the pre-receptive endometrium (PE) during embryo implantation^15^. Hence, we inferred that miR-449a potentially participates in regulating dynamic changes in goat uterine gene expression during the transition from the pre-receptive to the receptive phase. In addition, miR-449a possibly induces cell differentiation or apoptosis in a mutually exclusive fashion. While ensuring proper cell function, miR-449a plays roles in cancer prevention to mark a close connection between cell differentiation and tumor suppression^16^. By contrast, the reintroduction of miR-449a kills tumor cells efficiently or drives them into a permanent state of arrest called senescence^17^. Although miR-449 plays important roles in cell proliferation, differentiation and apoptosis, little is known about its potential role in RE development.

Leucine-rich repeat-containing G-protein coupled receptor 4 (LGR4), also called Gpr48, belongs to the leucine-rich GPCR (LGR) family^18,19^. Members of this family have multiple leucine-rich repeats at the N terminus and a seven-transmembrane domain^18^. LGR4 is a G protein-coupled receptor gene from an expressed sequence-tag database with high homology to glycoprotein hormone receptors, including the FSH receptor, LH receptor and TSH receptor genes^20^. LGR4 is widely expressed in multiple organs at both the embryonic and adult stages^21^, suggesting that it plays a vital role in growth retardation and renal and reproductive system development^22,23^. Furthermore, LGR4 contributes to uterine gland development, which supports decidualization during pregnancy in mice^24^. However, the function and molecular mechanism of LGR4 in the development of endometrial receptivity in the female goat remain unclear to date. LGR4 was predicted as a target gene of miR-449a using TargetScan (http://www.targetscan.org/), microRNA.org (http://www.microrna.org/) and miRanda (http://mirdb.org/) in both humans and mice.

Based on the specific profile of miR-449a during the implantation period, we hypothesized that miR-449a participates in embryo implantation by regulating RE development. In this study, we clarified the expression profile of miR-449a in PE and RE, and detected the influence of miR-449a on endometrial stromal apoptosis *in vitro*. In addition, this study suggested that miR-449a exerts an important impact on pinopode formation during embryo implantation *in vivo*. Our findings may provide an experimental basis for elucidating the miR-449a-related mechanisms involved in endometrial receptivity.

## Results

### Expression levels of miR-449a and LGR4 in the PE and RE

**Figure 1:**
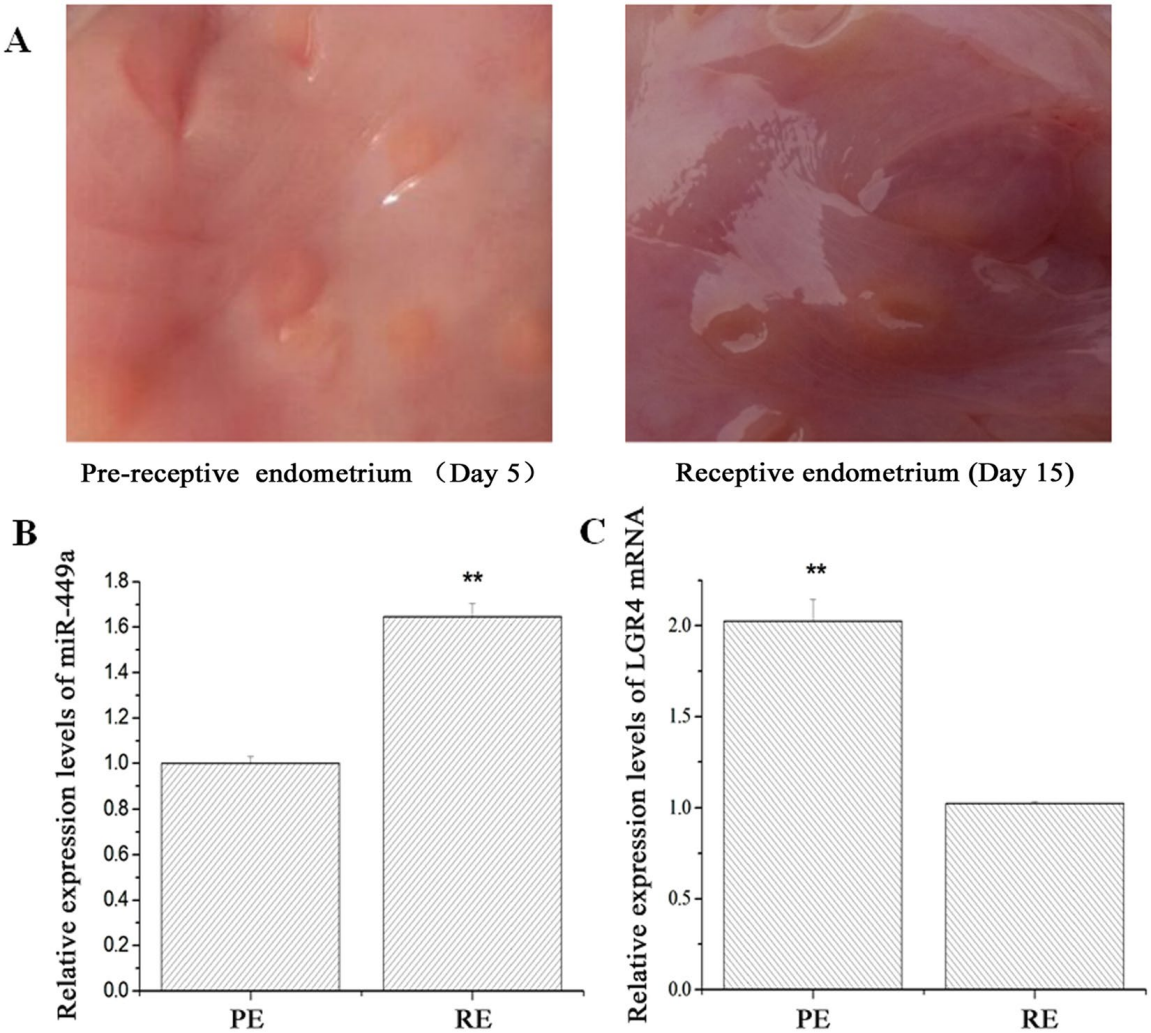
Expression levels of miR-449a and LGR4 mRNA in the pre-receptive and receptive endometria. (**A**) Surface of the pre-receptive (Day 5) and receptive endometria (Day 15). (**B**) Relative expression levels of miR-449a. (**C**) Relative expression levels of LGR4 mRNA. PE: Pre-receptive endometrium. RE: Receptive endometrium. The *GAPDH* gene or 18S rRNA was used for normalization. The experiment was independently repeated three times. The data are presented as the mean ± standard deviation. *P* < 0.01 is shown as **.

To determine the expression levels of miR-449a and LGR4, total RNAs were extracted from the PE and RE (Fig. 1A). On the 15th day of pregnancy, the endometrial surface thickened and became dark red. In addition, the caruncles on the endometrial surface increased on the 15th day. The RT-qPCR results showed that the expression levels of miR-449a were lower in PE (Fig. 1B) than in RE, which was consistent with previous sequencing data^15^. Additionally, the expression levels of the *LGR4* mRNA in the PE doubled compared with the levels in the RE (Fig. 1C). The results suggest that miR-449a plays a negative regulatory role in the expression of *LGR4* mRNA.

### MiR-449a directly targets the 3′UTR of the LGR4 mRNA

**Figure 2:**
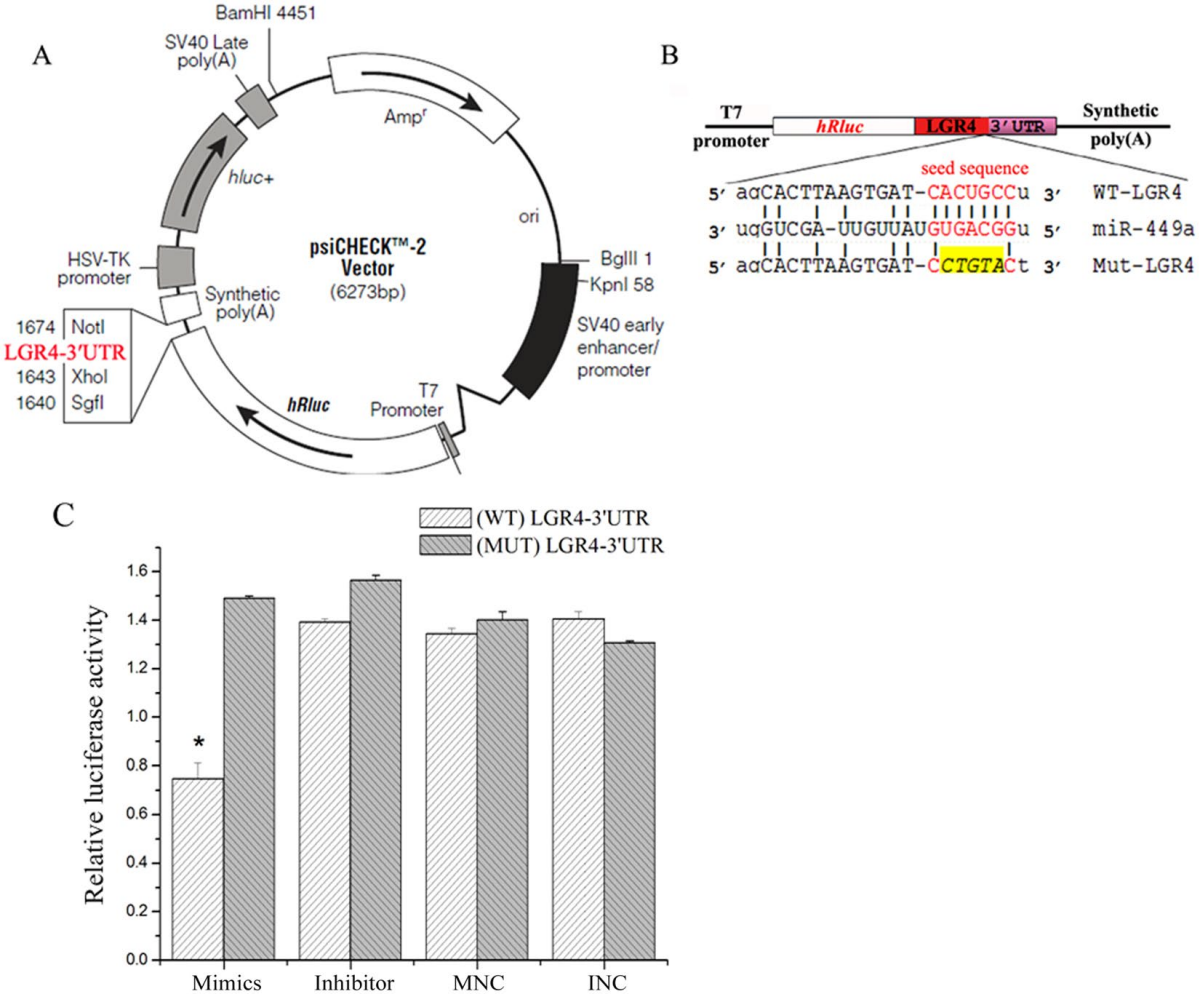
Identification of the miR-449a target gene. LGR4 gene was predicted as an important target. (**A**) psiCHECK-2 vector map (the insertion site of LGR4–3′UTR is marked in red). (**B**) Schematic of the design of the luciferase reporters with the WT-LGR4 3′UTR (WT-LGR4) or the site-directed mutant LGR4 3′ UTR (Mut-LGR4). The nucleotides in red represent the ‘seed sequence’ of miR-449a; the mutation nucleotides are marked in yellow. (**C**) The LGR4–3′UTR or its mutation luciferase reporter vectors were co-transfected with miR-449a mimics, mimic NC (MNC), miR-449a inhibitors or inhibitor NC (INC) into HEK293T cells. The experiment was independently repeated three times. Luciferase assays were performed 36 h after co-transfection. The data are presented as the mean ± standard deviation. *P* < 0.05 is shown as *.

To validate whether miR-449a directly targets the caprine *LGR4* gene through the predicted binding sites in the *LGR4* 3′UTR, the full-length 3′UTR containing miR-449a binding sites was cloned and inserted downstream of the luciferase gene in the psiCHECK-2 reporter plasmid (Fig. 2A). In addition, the mutated plasmid was constructed by inserting the LGR4 3′ UTR with the mutated miR-449a binding site (Fig. 2B). The wild-type (psiCHECK-LGR4-UTR-WT) or mutated (psiCHECK-LGR4-UTR-Mut) plasmids were co-transfected with miR-449a mimics, mimic NC (MNC), miR-449a inhibitors or inhibitor NC (INC) into HEK293T cells, respectively. Thirty-six hours after transfection, the luciferase activity of the miR-449a mimic group was significantly lower than that of the mimic NC group (*P* < 0.05), and this reduction was rescued in the mutation group (Fig. 2C). Thus, our results indicate that the caprine *LGR4* gene is confirmed as the target of miR-449a.

### MiR-449a regulates LGR4 expression

**Figure 3:**
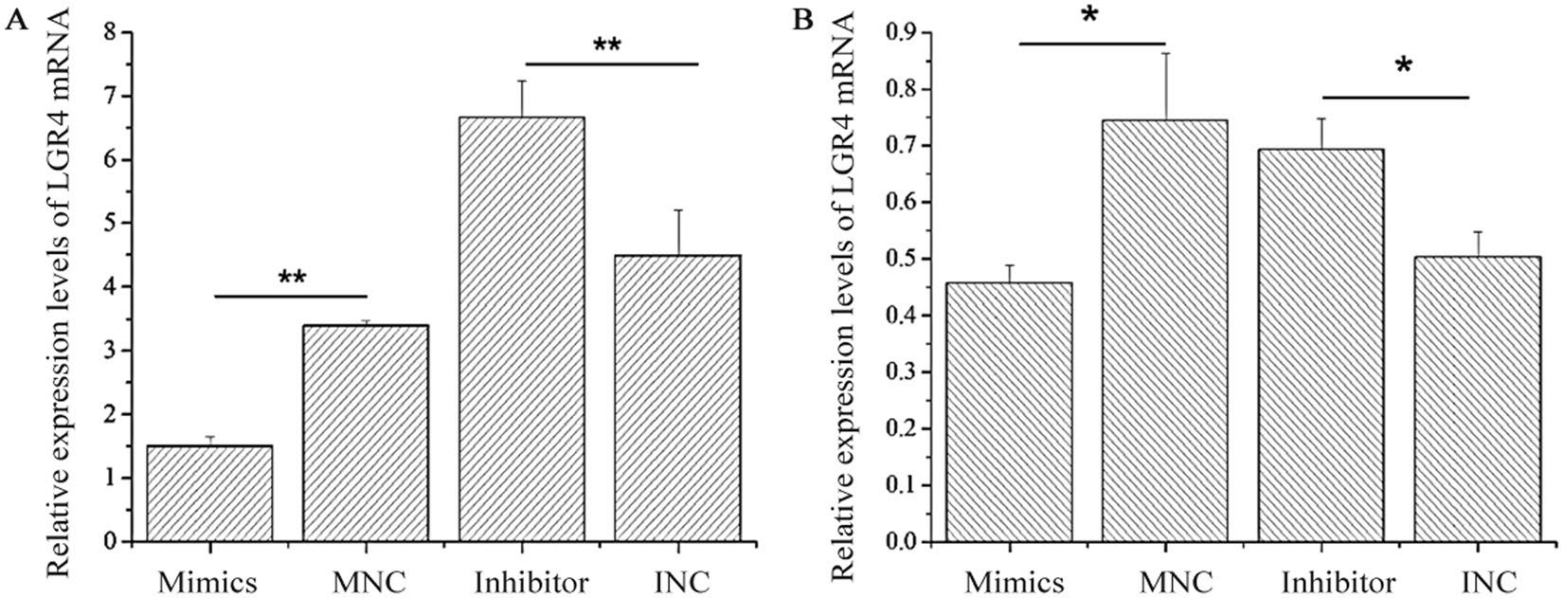
Effect of miR-449a on the LGR4 mRNA expression levels in ESCs. (**A**) Expression levels of LGR4 mRNA were detected 24 h (**A**) and 48 h after transfection (**B**). *GADPH* expression was used as a loading control. The experiment was independently repeated three times. The data are presented as the mean ± standard deviation. *P* < 0.05 is shown as *, and *P* < 0.01 is shown as **.

**Figure 4:**
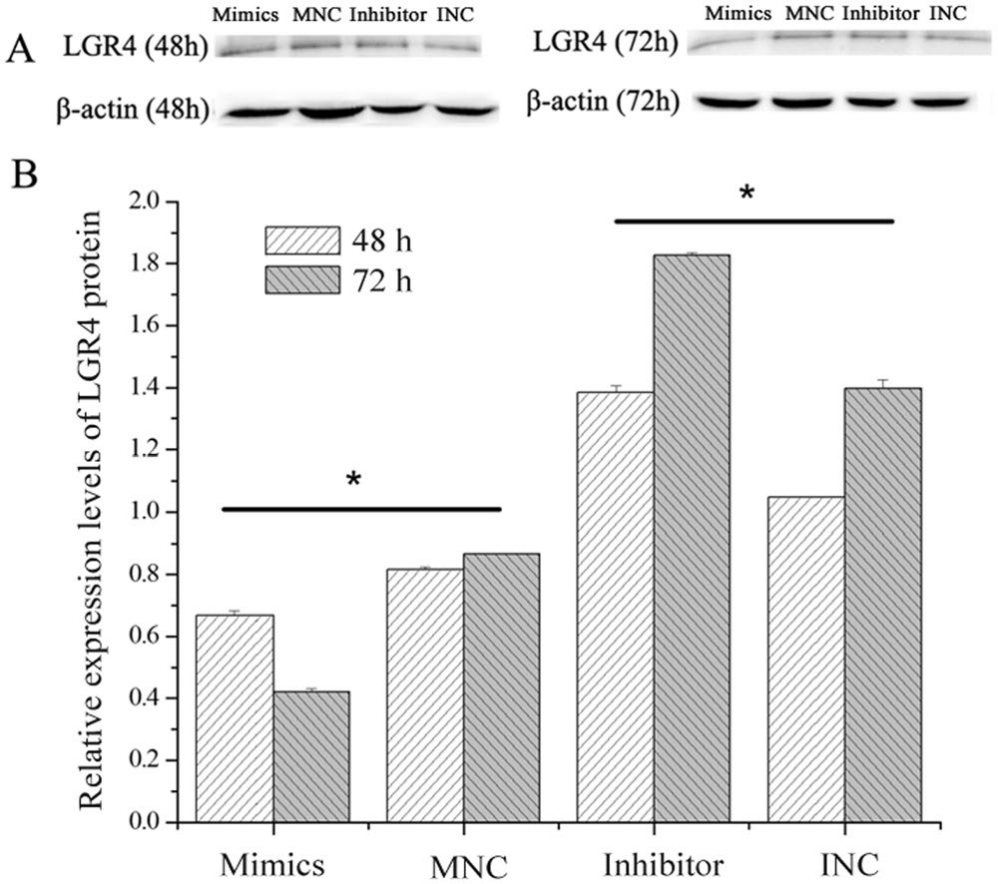
Effect of miR-449a on the LGR4 protein expression levels. (**A**) Western blot results. (**B**) Densitometric quantification of western blot results. Protein levels were normalized to β-Actin. The experiment was independently repeated three times. The data are presented as the mean ± standard deviation. *P* < 0.05 is shown as *.

After confirming the high abundance of miR-449a and low levels of *LGR4* mRNA in the REs of goats, we further investigated whether miR-449a down-regulates the expression levels of LGR4 during the ‘window of implantation,’ which may provide insights into the potential function of miR-449a in the regulation of the endometrium. We transfected ESCs with miR-449a mimics, MNC, miR-449a inhibitor and INC using Lipofectamine 2000. The results demonstrated that the expression levels of *LGR4* mRNA in ESCs markedly decreased after transfection for 24 h (Fig. 3A; *P* < 0.01) and 48 h (Fig. 3B; *P* < 0.05) in the miR-449a mimic and INC groups. The western blot results showed that the expression levels of the LGR4 protein were significantly reduced in the miR-449a mimic group compared with the MNC group at both 48 and 72 h (Fig. 4; *P* < 0.05). In contrast, the miR-449a inhibitors significantly increased the expression levels of the LGR4 protein at both 48 and 72 h (*P* < 0.05).

### MiR-449a regulates cell proliferation, cell cycle progression and apoptosis

**Figure 5:**
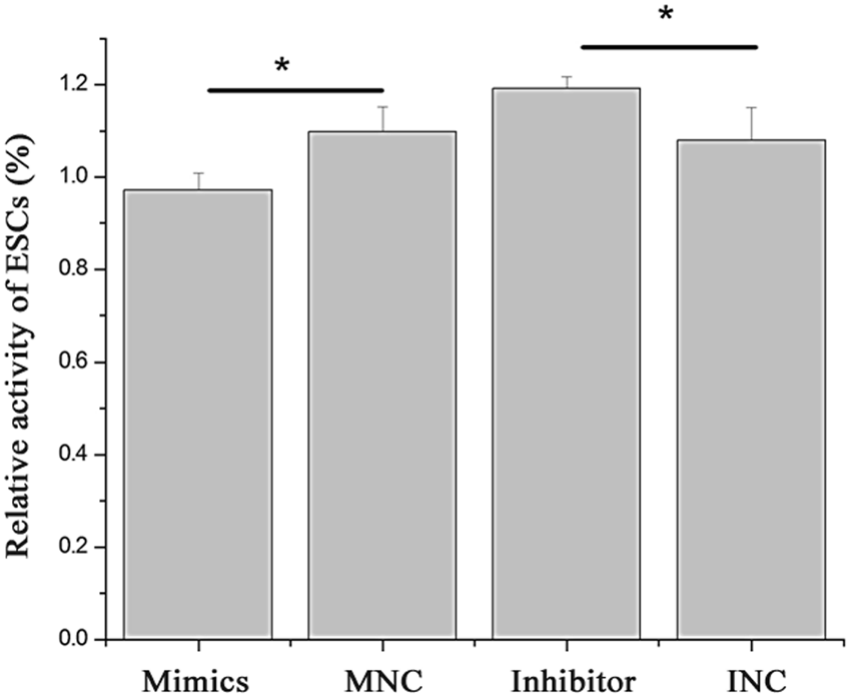
Effect of miR-449a on ESC metabolic activity. ESCs were transfected with miR-449a mimics, mimic NC (MNC), miR-449a inhibitors or inhibitor NC (INC). The relative activity of the ESCs was normalized to the absorbance values of the control cells (set to 100%) from three independent culture experiments. The data are presented as the mean ± standard deviation. *P* < 0.05 is shown as *.

**Figure 6:**
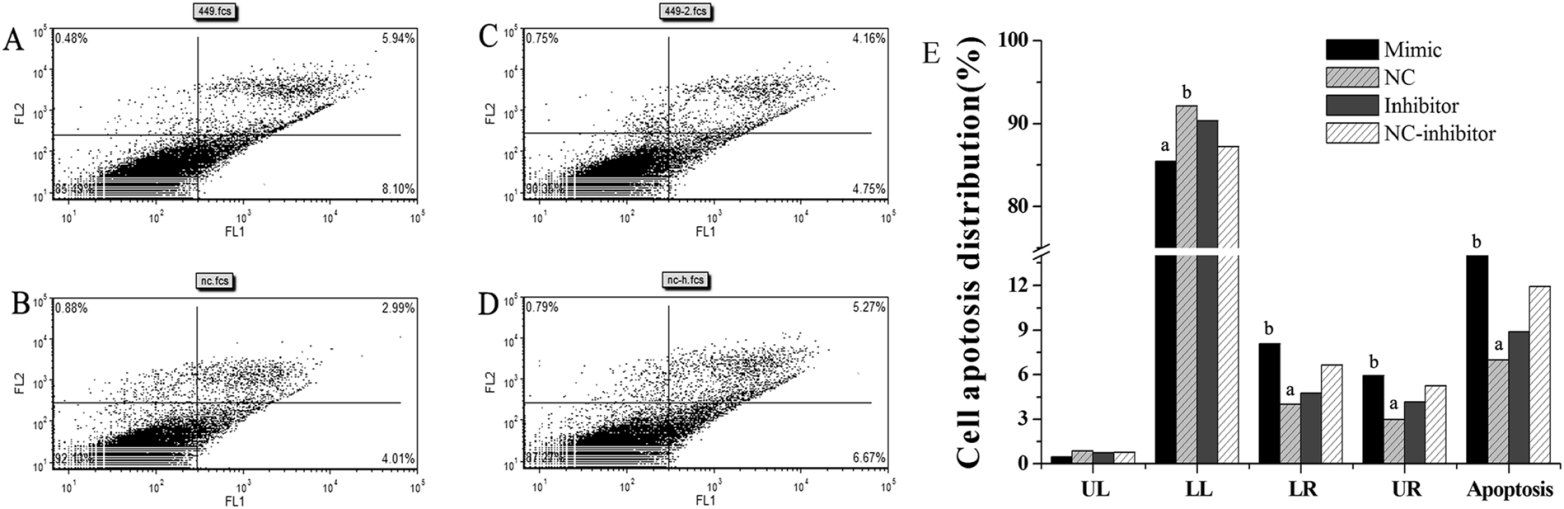
MiR-449a mediates ESC apoptosis. (**A**) ESCs transfected with miR-449a mimics. (**B**) ESCs transfected with mimic NC. (**C**) ESCs transfected with miR-449a inhibitors. (**D**) ESCs transfected with inhibitor NC. (**E**) UL represents necrotic cells; LL represents normal cells; LR represents early apoptotic cells; UR represents late apoptotic cells; Apoptosis represents early and late apoptotic cells. The experiment was independently repeated three times. The data are presented as the mean ± standard deviation. Different lowercase letters represent a significant difference at *P* < 0.05.

MiR-449a regulates cell metabolic activity, cell cycle progression and apoptosis in ESCs. MTT assays showed that miR-449a mimics significantly inhibited the activity of ESCs (Fig. 5; *P* < 0.05). Cell proliferation is generally regulated by progression through the cell cycle^25^. Consequently, the disruption of the cell cycle is considered a common cause of cell proliferation inhibition. To determine whether the anti-proliferative effect of miR-449a is due to cell cycle disruption, flow cytometry was used to analyze the changes in the cell cycle in ESCs. MiR-449a mimics significantly reduced the G_1_ cell population from 52.56% (MNC) to 42.19% with a concordant increase in the G2 and S phases from 47.44% (MNC) to 57.81% (Fig. S1), suggesting that miR-449a caused ESC cell cycle arrest. To confirm the effects of miR-449a on ESC apoptosis *in vitro*, cultured ESCs were transfected with miR-449a mimics or inhibitors. The apoptosis rate of the ESCs transfected with the miR-449a mimics or inhibitors was examined by flow cytometry (Fig. 6A–D). The number of apoptotic cells was significantly higher in the miR-449a mimic group (*P* < 0.05) than in the MNC group (Fig. 6E).

### Changes in uterine tissue structure

**Figure 7:**
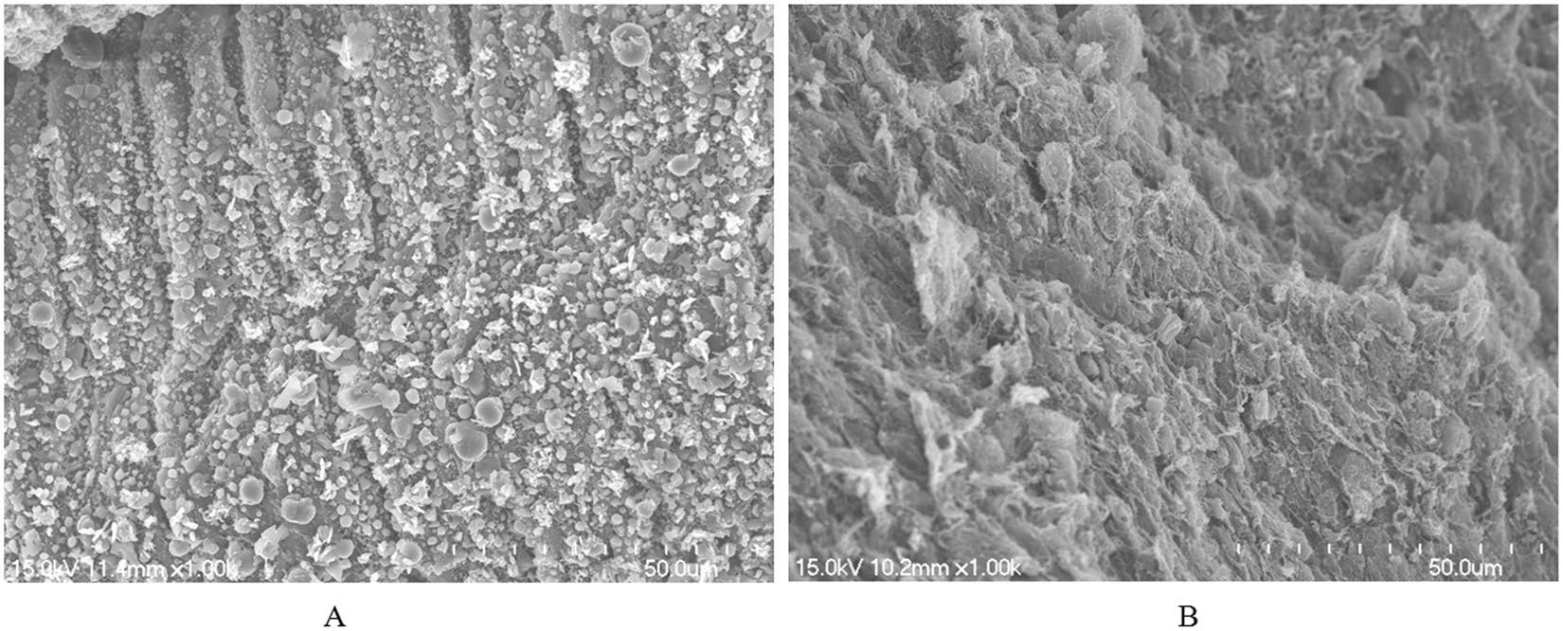
Surface topography of the endometrium under a scanning electron microscope. (**A**) Mice were injected with the miR-449a agomir. (**B**) Mice were injected with the miR-449a antagomir.

**Figure 8:**
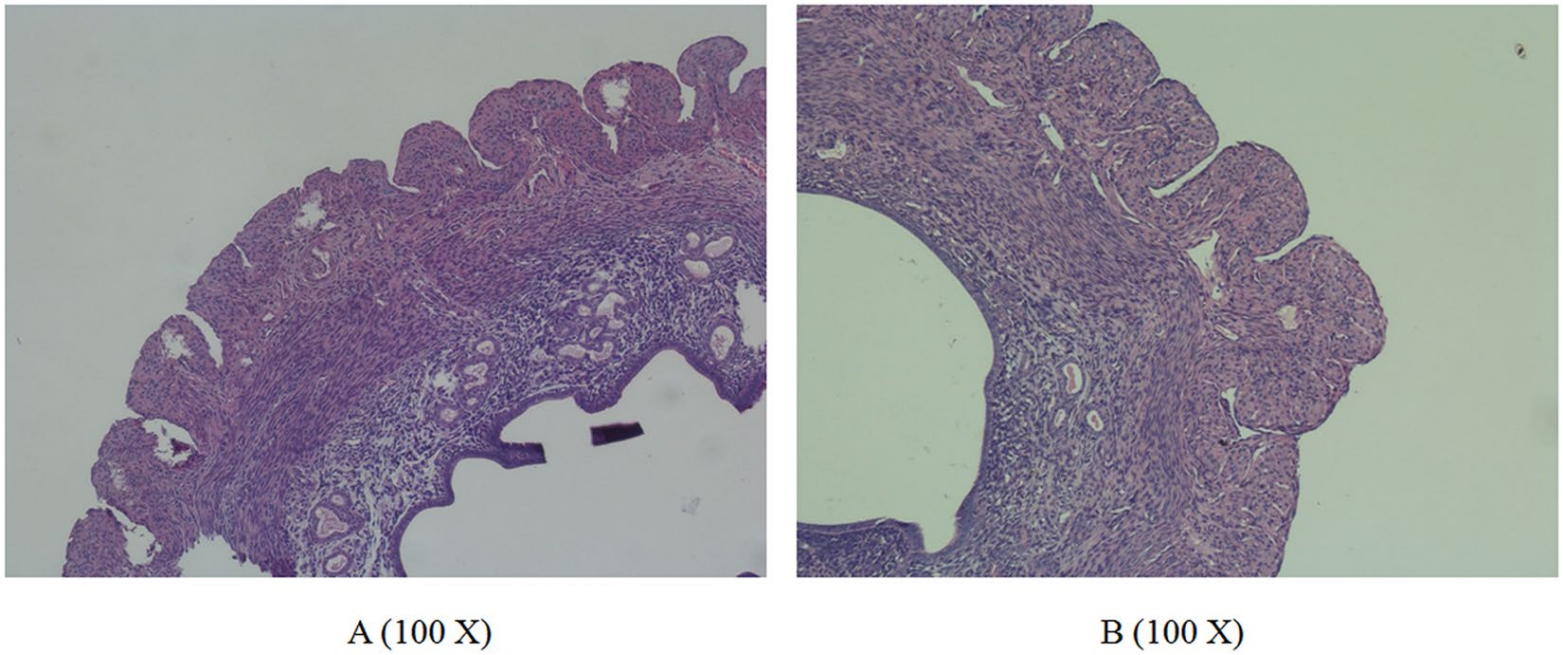
Surface topography of the endometrium under a biological microscope. (**A**) Mice were injected with the miR-449a agomir. (**B**): Mice were injected with the miR-449a antagomir.

Figure 7 Shows a scanning electron microscope (SEM) image of the surface topography of the endometrium. Rich pinopodes were observed on the endometrial surface in the miR-449a agomir group compared with the miR-449a antagomir group, suggesting that the endometrium was preparative for embryo implantation. The HE results showed that the endometrial thickness significantly increased in the miR-449a agomir group compared with the miR-449a antagomir group (Fig. 8).

## Discussion

For the acquisition of uterine receptivity, the following conditions need to be satisfied in the uterine environment: an endometrial preparation with stromal proliferation and epithelial differentiation in the pre-receptive phase and proper interactions between the uterus and the blastocyst later in the phase^6^. Endometrial receptivity is a dynamic process that is believed to be under post-transcriptional regulation because many genes expressed in the endometrium are epigenetically regulated^26^. For example, a previous study^27^ compared predicted miRNA target genes and messenger RNA microarray data and found 12 genes (e.g., CAST, CFTR, FGFR2 and LIF) that are important in endometrial receptivity. A recent study^28^ has revealed that miR-223-3p suppresses pinopode formation and LIF protein expression, which may lead to diminished embryo implantation. MiR-199a inhibits the angiogenic potential of endometrial stromal cells under hypoxia by targeting the HIF-1α/VEGF pathway^29^.

Computational predictions of miRNA targets are available but do not always reflect the *in vivo* situation. To avoid this issue, this study performed a luciferase assay to identify a miR-449a binding site in the 3′UTR of the *LGR4* mRNA. Initial reports posited that the extent of complementarity between the miRNA and its target mRNA governs either translational mRNA cleavage or repression^30^. In plants, nearly perfect complementarity leads to the degradation of target miRNAs^31^, whereas in animals, partial complementarity results in a translational block. However, some studies have reported that miRNAs can also induce mRNA degradation in animals and, conversely, translational repression in plants^32^. Furthermore, the state of the complementarity between the target mRNA and miRNA possibly affects mRNA degradation^33,34^. The results presented in the current study demonstrated that miR-449a decreased LGR4 protein production by altering *LGR4* mRNA levels. The uterine phenotype of the LGR4-null mouse may imply that the LGR4 orphan receptor plays a role in endometrial function and/or in endometrial disorders^35^. The mRNA expression of orphan receptors LGR4 and LGR5 in the human cyclic endometrium implies that the endometrium is potentially influenced by unknown mediators, which are possibly involved in fertility control^36^. Progesterone failed to inhibit luminal epithelial cell proliferation in the uteri of female LGR4-conditional KO mice, and the results of RT-qPCR and immunohistochemical analyses showed down-regulated progesterone signaling, which demonstrated that LGR4 is essential for the acquisition of endometrial receptivity through ovarian hormone signaling^37^.

In many mammals, endometrial cells are remodeled by apoptosis and cell proliferation throughout the estrous cycle^38^. Both cell proliferation and apoptosis (programmed cell death) are active in the bovine endometrium at the follicular and early luteal stages^39^. Some researchers believe that miR-449a prevents the G1-phase cell from entering the S phase by down-regulating several cell cycle regulators, such as cyclin-dependent kinases (CDKs) CDK6 and CDK4, cyclins (cyclin D1, Cyclin E2), the CDK-regulating cdc25 family of phosphatases (which activate CDKs by dephosphorylating the active site), geminin and the E2F transcription factors E2F1, E2F3 and E2F5^17^. The present results suggested that miR-449a provoked cell cycle arrest, which was consistent with the MTT assay results showing that miR-449 inhibited the proliferative ability of ESCs. Aside from regulating cell proliferation, miR-449 can also induce apoptosis^40^. MiR-449a induces cell apoptosis by regulating key factors in cell cycle and apoptosis. These factors include cyclin D1^41^, cell division cycle 25 homolog A^42^ and CDK6^43^. Animal studies noted that apoptosis of ESCs plays a major role in the establishment of a pregnancy^44,45^. In addition, decidualization, which is necessary for proper embryo implantation, allows endometrial stromal cells to induce apoptosis in reaction to embryonic stimuli *in vitro*; this phenomenon indicates that ESC apoptosis is an important process during implantation *in vivo* ^46^. *In vitro*, the present study showed that miR-449a could induce ESC apoptosis, however, miR-449a made endometrium thicker in the goat endometrium on gestational day 5 compared to gestational day 15 *in vivo*. There are differences between *in vitro* and *in vivo* experiments. *In vivo*, cell apoptosis and proliferation are dynamic processes. These results suggest that there are other factors that take part in this regulation process in the endometrium of dairy goats *in vivo*, although increased miR-449a induces ESC apoptosis *in vitro*. All these results suggest that there are huge and complicated regulatory networks of miR-449a in the endometrium of dairy goats *in vivo*, which needs further and more studies. *In vivo*, rich pinopodes were observed on the endometrial surface in the miR-449a agomir group compared with the miR-449a antagomir group. The RT-qPCR analysis showed that the expression levels of miR-449a in the PE were lower than the levels in the RE. Additionally, the expression levels of the LGR4 mRNA in the PE increased twice compared with the levels in the RE. These results indicate that miR-449a could increase endometrial receptivity.

In conclusion, we demonstrated that miR-449a could provoke cell cycle arrest and promote ESC apoptosis. Observation by SEM revealed that miR-449a increased the number of pinopodes on the endometrial surface *in vivo*. The HE staining demonstrated that the endometrial thickness was also significantly increased in the miR-449a agomir group compared with the miR-449a antagomir group. Our findings may provide relevant data for studies on the miRNA-mediated regulation of endometrial receptivity.

## Materials and Methods

### Ethics statement

All animals were maintained in accordance with Proclamation No. 5 of the Ministry of Agriculture, China. Sample collection was approved by the Institutional Animal Care and Use Ethics Committee of Northwest A&F University and performed in accordance with the ‘Guidelines for Experimental Animals’ of the Ministry of Science and Technology (Beijing, China). The Institutional Animal Care and Use Ethics Committee of Northwest A&F University approved this study.

### Tissue collection

A total of 10 healthy 24-month-old multiparous Xinong Saanen dairy goats (Xinong Saanen) were induced for estrous synchronization for this study. The first day of mating was considered to be day 0 of pregnancy. Gestational days 5 and 15 are important time points for embryo implantation in goats^47^. The experimental goats were observed three times daily to ascertain estrous signs and then mated naturally twice during the estrous cycle. The goats were euthanized upon losing consciousness after intravenous injections of barbiturates (30 mg/kg) on gestational day 5 (PE) and gestational day 15 (RE). Endometrial samples from 5 goats at gestational day 5 and from 5 goats at gestational day 15 were obtained from the anterior wall of the uterine cavity^48^. All tissue samples were washed briefly with phosphate buffered saline (PBS) and then immediately frozen in liquid nitrogen.

### Target gene prediction of miR-449a

The target genes of miR-449a were screened using TargetScan (http://www.targetscan.org/index.html). This software predicts mammalian miRNA target genes by combining a thermodynamic model of RNA interaction and sequence alignment analysis to detect conserved miRNA binding sites. The TargetScan algorithm searches the UTR of the mRNA for segments of perfect Watson–Crick complementarily to bases 2–8 of the miRNA (numbered from the 5′ end and referred to as the miRNA seed) and extends each seed match with additional complementary base pairs as far as possible in each direction, allowing G-U pairs while stopping at mismatches, and assigns a folding-free energy to each miRNA–target site interaction^49^. Other bioinformatic analyses of the miRNA binding sequences in the 3′UTRs of target genes were performed to identify a miRNA that could bind to the 3′UTR of the LGR4 mRNA.

### Luciferase assay

Fragments of the LGR4 3′UTR containing the predicted LGR4 binding site were cloned with the following primers: F: 5′-GC***CTCGAG***TGTTTGTAACTGTTTCTCCCAT-3′ and R: 5′-GC***GCGGCCGC***CCAGGCTCCTCCATCCAT-3′. Italicized and bold letters indicate *Xho*I and *Not*I endonuclease restriction sites. Fragments of the LGR4 3′UTR were then linked to a pMD19-T vector using a TA Cloning Kit (Invitrogen, CA, USA). The recombinant pMD19-T vectors were digested by *Xho*I and *Not*I endonucleases. Finally, the digested products were inserted between the *Renilla* and firefly luciferase genes in a psiCHECK-2 vector (Promega, WI, USA). In addition, the mutated plasmid was constructed by inserting the LGR4 3′ UTR with the mutated miR-449a binding site between the *Xho*I and *Not*I sites.

Human embryonic kidney 293 T (HEK293T) cells were plated in 24-well plates at 60% confluence. Cells were then co-transfected with the LGR4 3′UTR or its mutant reporter construct, together with the miR-449a mimics, mimic NC, miR-449a inhibitors or inhibitor NC. The miR-449a mimics, inhibitors and NC were chemically synthesized and purified by Shanghai GenePharma (Shanghai, China). Transfections were performed with Lipofectamine 2000 transfection reagent (Invitrogen) following the manufacturer’s instructions. Cell lysates were harvested by direct lysis after 36 h of culture. Luciferase activity was measured in triplicate using the Dual Luciferase Assay System (Promega). *Renilla* luciferase activity was normalized to firefly luciferase activity. Each experiment was independently repeated three times.

### Cell culture and transfection

Caprine endometrial stromal cell (ESC) lines were provided by Dr. Yaping Jin (Northwest A&F University, Yangling, China). These cells were immortalized by transfection with the human telomerase reverse transcriptase^50^. Cells were cultured and maintained in Dulbecco’s Modified Eagle’s Medium (DMEM)-Hank F12 (Gibco, Invitrogen Corporation, Grand Island, NY, USA) supplemented with 10% charcoal-stripped fetal calf serum (FCS; Atlanta Biologicals, Lawrenceville, GA, USA) and antibiotics (100 U/mL penicillin and 100 mg/mL streptomycin; Gibco, Invitrogen Corporation, Grand Island, NY, USA) at 37 °C in 5% CO_2_ atmosphere. The medium was changed 24 h after seeding and then at 48-h intervals until the cultures achieved subconfluence. Subconfluent cultures of ESCs were subjected to trypsinization, sedimentation by centrifugation, and resuspension in DMEM/F12 + 10% charcoal-stripped FCS. ESCs were plated at a density of 7.5 × 10^5^ in six-well plates. Then, 50%-confluent cells were transfected with miR-449a mimics, mimic NC, miR-449a inhibitors and inhibitor NC using the X-tremeGENE siRNA transfection reagent (Roche, Basel, Switzerland) in accordance with the manufacturer’s protocols.

### RNA isolation, reverse transcription and RT-qPCR

Total RNA was extracted from PE, RE and ESCs that were transfected with miR-449a mimics, mimic NC, miR-449a inhibitors and inhibitor NC by using TRIzol reagent (TaKaRa, Dalian, China) in accordance with the manufacturer’s instructions, respectively. RNA concentration and purity were determined by measuring the optical density (OD) at wavelengths of 260 and 280 nm with an Epoch microplate spectrophotometer (BioTek Instruments, Inc., USA). The OD_260/280_ ratios were >1.8 and <2.1 for all samples. Total RNA (500 ng) was used to convert mRNAs into cDNAs using the PrimeScript RT reagent Kit (TaKaRa, Dalian, China) in accordance with the manufacturer’s instructions.

RT-qPCR was then performed in a 25 μL reaction volume containing 12.5 μL of SYBR Premix Ex Taq II (TaKaRa, Dalian, China), 2 μL of template cDNA and 1 μM primers using the CFX Connect Real-Time PCR Detection System (Bio-Rad, CA, USA). The thermal cycling conditions were 95 °C for 10 min, followed by 40 cycles at 94 °C for 15 s, 60 °C for 30 s and 72 °C for 30 s. Primers are shown in Table 1 and S1. The *GAPDH* gene or 18S rRNA was used for normalization. Each experiment was independently repeated three times, and the fold change in the expression of each gene was analyzed via the 2^−ΔΔCt^ method.

**Table 1:** . Primer information for RT-qPCR.

Gene name	Primer sequence (5′-3′)	Tm (°C)
*LGR4*	F: AGTTTCCATCAGCAGCCAAG	59
	R: GCAGCAGTCACACACAGTCA	
*GADPH*	F: ACTTTGGCATCGTGGAGG	60
	R: GAAGAGTGAGTGTCGCTGTTG	

### Protein extraction and western blot analysis

Protein extraction from ESCs was performed as previously described. In brief, cells grown to confluence were harvested and lysed in RIPA buffer (50 mM Tris–HCl, 150 mM NaCl, 1% SDS, 0.5% sodium deoxycholate, 1 mM DTT, 100 mM PMSF, 1%NP-40 and 1 × protease inhibitor cocktail; Roche, Indianapolis, IN, USA). After standing for 30 min at 4 °C, the mixture was centrifuged for 10 min (11,000 × *g*). Soluble protein in the supernatants was collected. The total protein concentration was determined using the Bio-Rad DC Protein Assay, and the supernatants were then diluted with gel loading buffer (Beyotime, Shanghai, China) and boiled for 8 min. Protein (30 mg) from each treatment was subjected to 12% SDS-polyacrylamide gel electrophoresis and then transferred onto nitrocellulose membranes (Millipore, Bedford, MA, USA) at 100 V for 1.5 h in an ice bath. Non-specific binding sites were blocked with 5% fat-free powdered milk (blocking solution) and Tris-buffered saline [10 mM Tris-HCl (pH 7.4), 0.5 M NaCl] plus Tween 20 [0.2% (vol/vol)] (TBST) at room temperature for 1 h. After three washes for 10 min each with TBST, the membranes were incubated overnight at 4 °C with rabbit monoclonal antibodies against goat LGR4 (Santa Cruz Biotechnology, USA) at 1: 200 dilutions. β-Actin (Beyotime, Shanghai, China) antibody was used as the internal loading control at a 1:1000 dilution following the same procedure described above. After this incubation, the membranes were washed three times and incubated at room temperature for 2 h with horseradish peroxidase-conjugated goat anti-rabbit IgG (Beyotime, Shanghai, China) at a 1:1000 dilution. After three washes for 5 min each with TBST, proteins were detected by enhanced chemiluminescence (Advansta, California, USA). Quantification was performed using the Quantity One program (Bio-Rad, California, USA).

### MTT assay

The methyl thiazolyl tetrazolium (MTT) (Sigma, St. Louis, MO, USA) colorimetric assay was used to screen cell viability. The cells were seeded into 96-well plates at a density of 2 × 10^3^ cells/well and then transfected with miR-449a mimics, mimic NC miR-449a inhibitors or inhibitor NC. After incubation at 37 °C in a 5% CO_2_ incubator for 24 h, the absorbance was measured at a wavelength of 490 nm as previously described^51^. All experiments were performed in triplicate and repeated three times to ensure the reproducibility of the results.

### Analysis of cell cycle

The mechanism of cell growth deficiency in miR-449a-transfected ESCs was evaluated using a cell cycle staining kit (Liankebio, Hangzhou, China) in accordance with the manufacturer’s instructions. The cells were harvested after 48 h, washed three times with cold PBS and fixed in 70% ethanol in PBS at −20 °C for 24 h. After fixation, the cells were labelled with 0.5 mL of propidium iodide (PI) staining buffer containing 100 µg/mL RNase A and 50 µg/mL PI at 37 °C for 30 min in the dark. Analyses were performed with a BD LSR flow cytometer (BD Biosciences, San Diego, CA).

### Analysis of cell apoptosis

Cell apoptosis analysis was carried out using an Annexin V-FITC/PI apoptosis kit (Liankebio, Hangzhou, China) in accordance with the manufacturer’s instructions. After being treated with miR-449a mimics, mimic NC, miR-449a inhibitors or inhibitor NC for 48 h, ESCs were collected and incubated with Annexin V-FITC and PI at room temperature for 10 min in the dark. Annexin V-positive and PI-negative cells were defined as early apoptotic cells. The late apoptotic cells were Annexin V- and PI-positive cells. Analyses were performed with a BD LSR flow cytometer (BD Biosciences).

### Detection of uterine tissue structure

Eight estrous female mice (7-week-old; C57BL6) induced by pregnant mare serum gonadotropin and human chorionic gonadotropin were used. After mating with four male mice, the day when a vaginal plug was observed was considered as embryonic development day 0. Mice were treated with miR-449a agomir (F: 5′-UGGCAGUGUAUUGUUAGCUGGU-3′ and R: 5′-CAGCUAACAAUACACUGCCAUU-3′) or antagomir (5′-ACCAGCUAACAAUACACUGCCA-3′) at 60 h post-vaginal plug. The antogimir was modified with two phosphorothioates at 5′ end, four phosphorothioates and cholesterol at 3′ end. Antisense agomir was synthesized with same chemical modifications as antogimir. The agomir and antagomir were diluted with 0.1% diethylpyrocarbonate (DEPC) H_2_O. (1) For experimental group one, four mice were injected with the miR-449a agomir by caudal vein injection (6 mg/kg body weight). (2) For control group two, four mice were injected with the miR-449a antagomir (6 mg/kg body weight). Uterus specimens were collected at 96 h post-vaginal plug and then fixed with 2.5% glutaraldehyde or a 10% formalin neutral fixative.

Glutaraldehyde-fixed (2.5%) samples were thoroughly washed with PBS buffer, dehydrated in graded ethanol, placed in 2% isoamyl alcohol for 3 h and subjected to critical point drying. The samples were attached to the sample stage for observation with the surface (endometrial cavity surface) facing up and then painted with silver conductive plastic using a vacuum coating apparatus for coating metal samples. Then, the samples were observed under an aJSM-6330F SEM (JEOL, Japan); all results were descriptive and not quantified.

Tissue samples were fixed in a 10% formalin neutral fixative for 1 h, washed several times with ethanol (70%–100%) for 5.5 h and xylol (2.5 hours) and embedded into paraffin (2 h). Hematoxylin/eosin (HE) staining of 5-mm tissue sections was performed by automation (Gemini, Shandon Veristain, GMI, Inc. Minnnesota, USA) with deparaffinization with xylol for 10 min, washing with ethanol and water and subsequent staining with hematoxylin for 3 min and eosin for 20 s.

After the color reaction, sections were dehydrated through an ethanol series into xylene and mounted using the Permount mounting medium (Fisher Scientific, PA). The thickness of endometrium was analyzed through measuring the distance from medialis round muscles to intimal surface under semi-automatic inverted biological microscope with the ruler of imaging system (DMI4000B, Leica, Germany).

### Statistical analysis

All data were processed with SPSS 16.0 (SPSS Inc., Chicago, IL, USA). One-way ANOVA was used to compare the differences, and the least significant difference method was used for further analysis. The data are presented as the mean ± standard deviation. The differences were considered significant at *P* < 0.05 and very significant at *P* < 0.01.

## Electronic supplementary material

supplementary information
