# Supplementary material for: MiR-449a regulates caprine endometrial stromal cell apoptosis and endometrial receptivity
Source: Sci Rep. 2017 Sep 25;7:12248. doi: 10.1038/s41598-017-12451-y (PMC5612931; doi:10.1038/s41598-017-12451-y)

MiR-449a regulates caprine endometrial stromal cell apoptosis and endometrial receptivity

Xiaopeng An[1a], Xiaorui Liu[1a], Lei Zhang[1a], Junze Liu[a], Xinyan Zhao[b], Kaiwen Chen[a], Haidong Ma[a], Guang Li[a], Binyun Cao[a*], Yuxuan Song[a]**

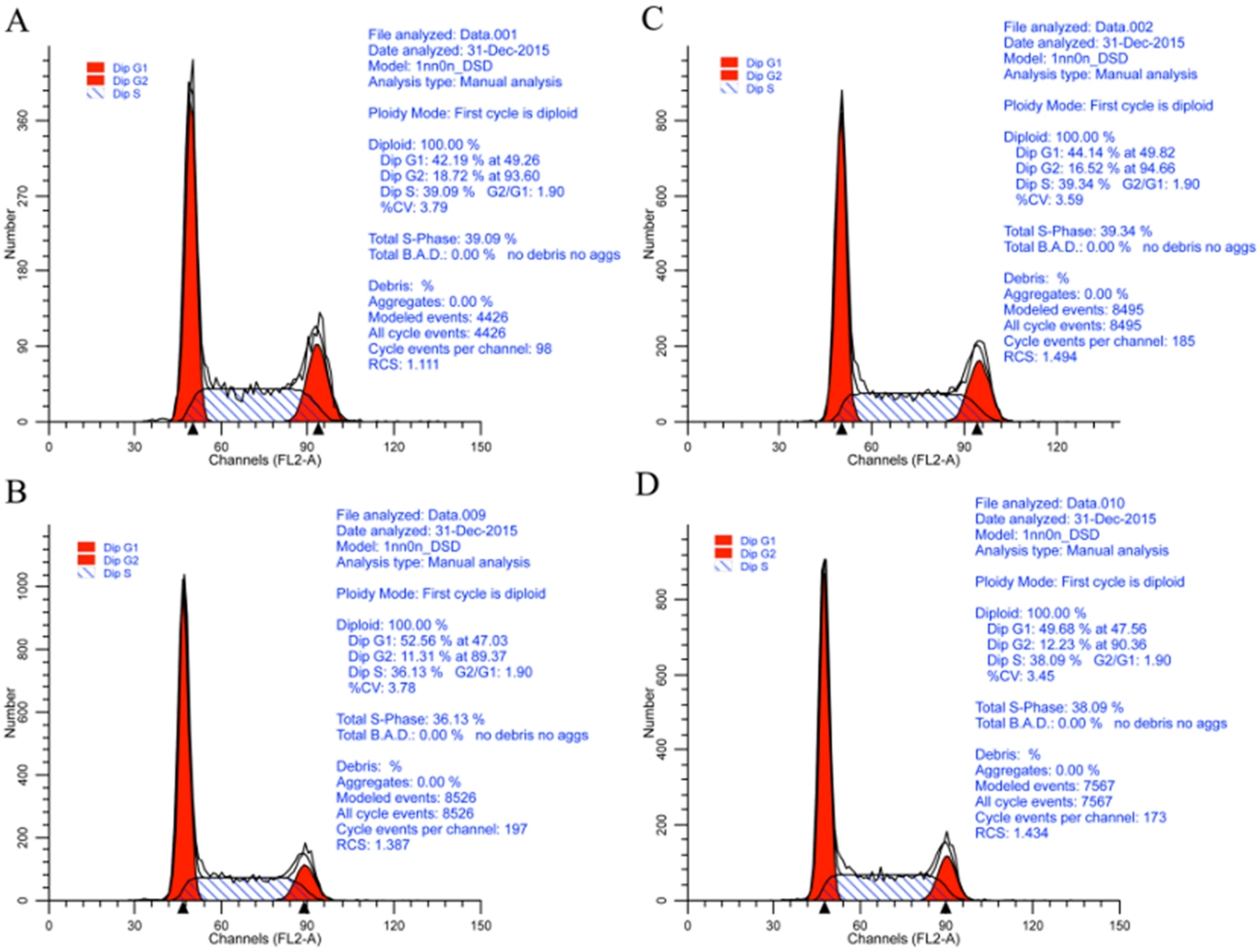

Figure S1 Effect of miR-449a on ESC cycle. A: ESCs were transfected with miR-449a mimics. B: ESCs were transfected with mimic NC (MNC). C: ESCs were transfected with miR-449a inhibitors. D: ESCs were transfected with inhibitor NC (INC).

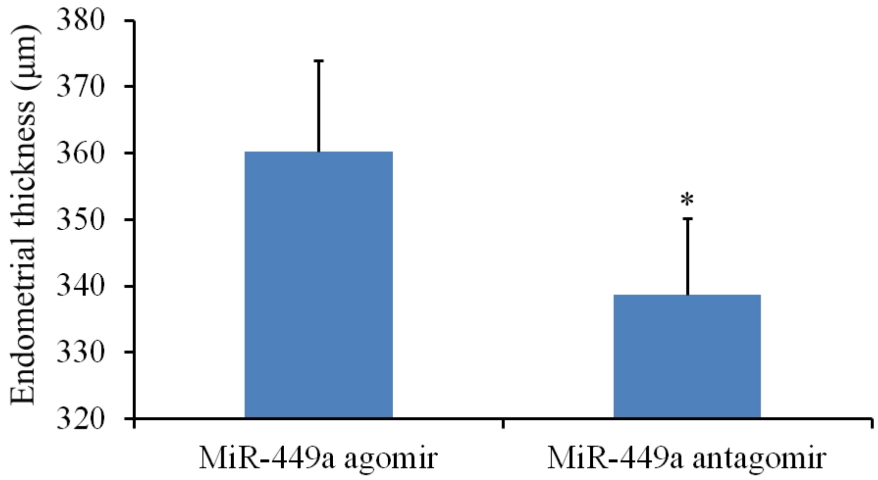

Figure S2 Endometrial thickness in the miR-449a agomir and antagomir groups. *P<0.05.

Table S1 Primer information of Stem-loop RT-qPCR for miR-449a

Name	Primer	Sequence (5'-3')	Tm (^{o}C)
miR-449a	RT-Primer	GTCGTATCCAGTGCAGGGTCCGAGGT ATTCGCACTGGATACGACACCAGCTA	60
	Forward primer	GCGCGCTGGCAGTGTATTGT	
	Reverse primer	GTGCAGGGTCCGAGGT	
18S rRNA	Forward primer	GTGGTGTTGAGGAAAGCAGACA	60
	Reverse primer	TGATCACACGTTCCACCTCATC	

Thank you for your question. They are on the same membranes. After transfer, the target protein and internal reference protein were separately cut from the transfer film according to molecular weight for primary and secondary-antibody incubation, thus the bands were obtained in two separated film for 48h and 72h. The gels were described in the figure legend. In the images of LGR (48h) and LGR (72), this is Mimics, MNC, Inhibitor and INC from left to right, and β-Actin (48h and 72h) are their control from left to right. The results are as follows:

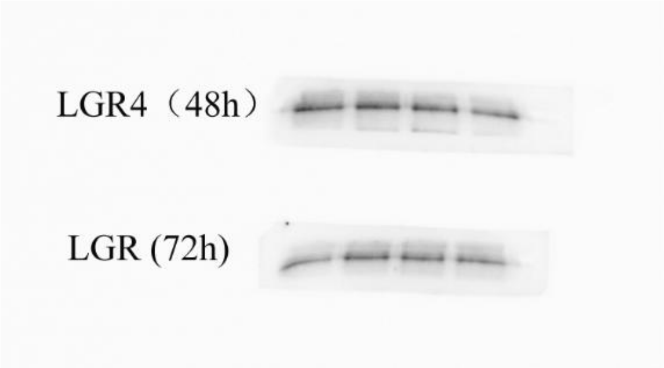

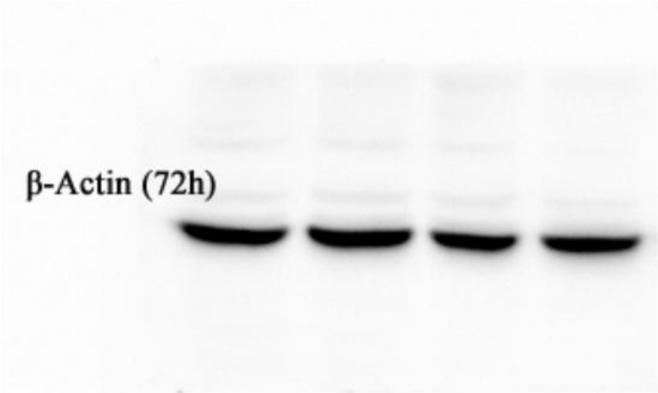

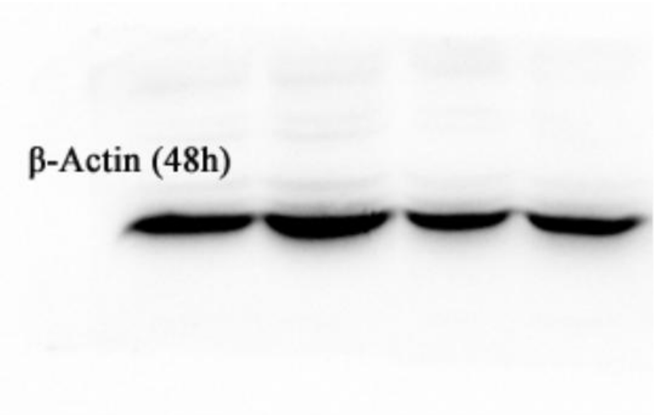

Supplement: Supplementary file 1 — supplementary information [file 41598_2017_12451_MOESM1_ESM.pdf]
